# Exosome crown proteins are promising markers for liquid biopsy of breast cancer

**DOI:** 10.3389/fonc.2026.1698043

**Published:** 2026-03-11

**Authors:** Svetlana Tamkovich, Aleksei Shefer, Andrey Kalichkin, Alyona Chernyshova

**Affiliations:** 1 Institute of Oncology and Neurosurgery, E.N. Meshalkin National Medical Research Center, Ministry of Health of the Russian Federation, Novosibirsk, Russia; 2 Institute of Medicine and Medical Technologies, Novosibirsk State University, Novosibirsk, Russia; 3 Institute of Chemical Biology and Fundamental Medicine, Siberian Branch of Russian Academy of Sciences, Novosibirsk, Russia; 4 Oncology Department, Novosibirsk State Medical University, Novosibirsk, Russia

**Keywords:** blood, breast cancer, exosomes, extracellular vesicles (EVs), liquid biopsy, MMPs, tetraspanins, tumor markers

## Abstract

Breast cancer (BC) remains the most common malignant disease in women. However, currently used instrumental and laboratory (CA15-3, CA125, etc.) diagnostic methods demonstrate insufficient sensitivity and specificity for early and reliable detection of BC. In this regard, great expectations are associated with the liquid biopsy method based on the identification of tumor cells or their components, including tumor-derived exosomes. The purpose of this study is to analyze current data on exosome proteins that can be used for diagnostics using liquid biopsy. This review discusses the role of exosomal crown proteins in the spread of BC and assesses their potential as diagnostic markers. The undoubted advantages of using exosomal crown proteins as tumor markers compared to other components of the tumor secretome are the simplicity and reproducibility of their analysis by flow cytometry, as well as, unlike microRNA, tissue specificity. In contrast to prior reviews that primarily catalogue extracellular vesicle cargo, we specifically assess surface-accessible proteins that combine biological relevance with analytical feasibility. This approach bridges mechanistic EV biology with the practical design of clinically translatable diagnostic assays. Standardization of protocols for exosome isolation, antibody validation, and signal amplification will be critical to the successful implementation of this approach into routine clinical practice. Integration of exosomal coronary protein profiling into modern oncology workflows may open new opportunities for early detection, long-term surveillance, and precision treatment of BC.

## 1 Introduction

Malignant neoplasms of the breast represent the most prevalent oncological pathology among women. According to the World Health Organization (WHO), in 2022, 2.3 million new cases of breast cancer (BC) were registered worldwide, with 666,000 disease-related deaths documented (1).

The challenge of timely BC detection stems not only from its frequently asymptomatic course but also from the absence of reliable biomarkers capable of identifying malignant transformation at early stages. Early detection contributes to improved effectiveness of anticancer therapy and significantly reduces mortality in affected patients (2). Nevertheless, according to data from a Russian cohort, more than one−quarter of newly diagnosed BC cases were detected at advanced (III–IV) stages (3), underscoring the ongoing challenges of early detection.

At present, BC diagnostics employ an integrated approach, including biopsy and various imaging modalities such as mammography, ultrasonography, magnetic resonance imaging (MRI), and positron emission tomography (PET). Mammography remains the standard diagnostic method, allowing detection of tumors as small as 10 mm in diameter; however, its use in longitudinal monitoring is limited due to radiation exposure (0.4 mSv) (4). Additional drawbacks include age-related restrictions (in women under 40 years, breast tissue tends to be denser), a high rate of false negatives in palpable tumors [up to 15% (4)], and a substantial incidence of false positives [up to 61% among women aged 40 to 50 years (5)]. Combining mammography with other imaging techniques may reduce the frequency of false-positive results (6).

Ultrasonography is also widely used for detecting malignant lesions. Key advantages of ultrasonography include the absence of ionizing radiation - rendering it safe for pregnant and lactating women, its repeatability, broad availability, and higher sensitivity in assessing dense breast tissue compared to mammography. Limitations of this method include its inability to detect microcalcifications (7) and significantly reduced sensitivity in breasts with a high adipose tissue content (8).

MRI lacks the above-mentioned limitations and enables accurate assessment of the size and localization of lesions as small as 5 mm, as well as detection of microcalcifications and residual malignancy in post-surgical patients (9). PET is another effective instrumental modality for BC diagnosis. However, in addition to sharing the limitations inherent to MRI, PET is unable to detect lesions smaller than 10 mm (8).

The sensitivity and specificity of biopsy - the principal confirmatory method in BC diagnostics depend on the quantity and purity of the sampled tissue, as well as the accuracy of sampling within the neoplasm (10). As a result, these parameters may vary considerably, ranging from 52% to 93% (11).

**Figure 1:**
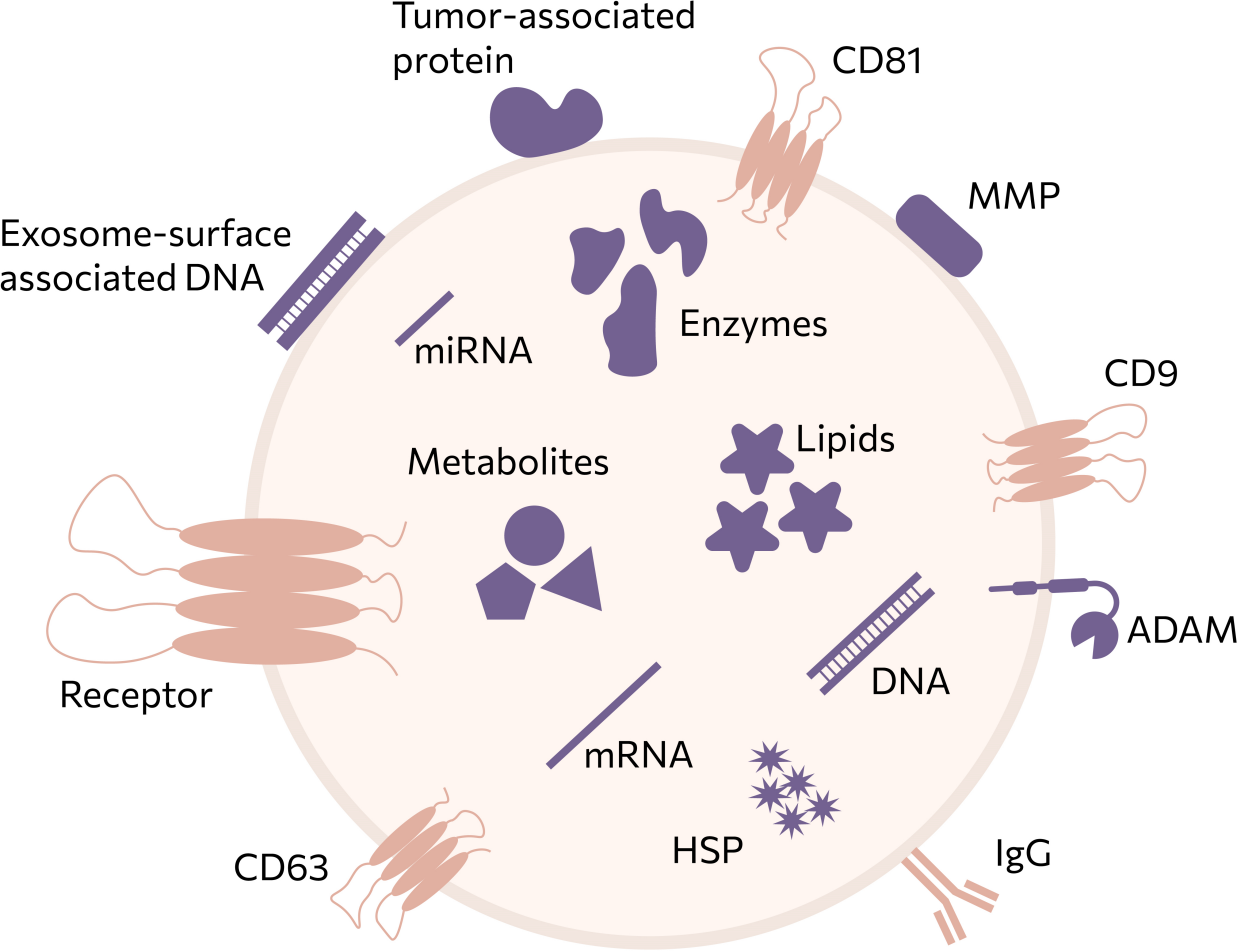
Main sources of markers for “liquid biopsy”.

Personalized medicine, which relies on characterizing the heterogeneous molecular profile of the tumor, currently offers the highest efficacy in cancer treatment. However, modern imaging techniques and tissue biopsy cannot fully capture this complexity. Diagnostic strategies involving “liquid biopsy” offer several advantages over conventional tissue biopsy, including minimal invasiveness, high sensitivity, suitability for serial blood sampling, and more accurate reflection of tumor heterogeneity (12). Another advantage of liquid biopsy is its ability to detect neoplasms in anatomically inaccessible regions (13) (**Figure 1**).

Biomarkers detectable via liquid biopsy include circulating tumor cells (CTC) (14), extracellular vesicles (EVs) including exosomes (15, 16) and nucleic acids circulating in blood as part of protein complexes, such as tumor DNA (17), RNA [including microRNAs (18), lncRNAs (19), circular RNA, and messenger RNAs (20)], and tumor-derived proteins (20). The high concentration of exosomes in biological fluids (10^8^ exosomes/mL versus ~10 tumor cells/mL) renders them a highly promising source of biomarkers for liquid biopsy (21). Despite the extensive literature describing exosome biogenesis and function, relatively few studies critically evaluate which exosomal surface proteins are truly suitable for routine liquid biopsy. The aim of this review is therefore to systematically assess exosomal corona proteins that are exposed on the vesicle membrane, functionally involved in tumor progression, and are compatible with standardized analytical platforms such as flow cytometry and immunoaffinity capture. This focused framework enables integration of fundamental EV biology with clinically oriented diagnostic strategies.

## 2 Features of exosome circulation

**Figure 2:**
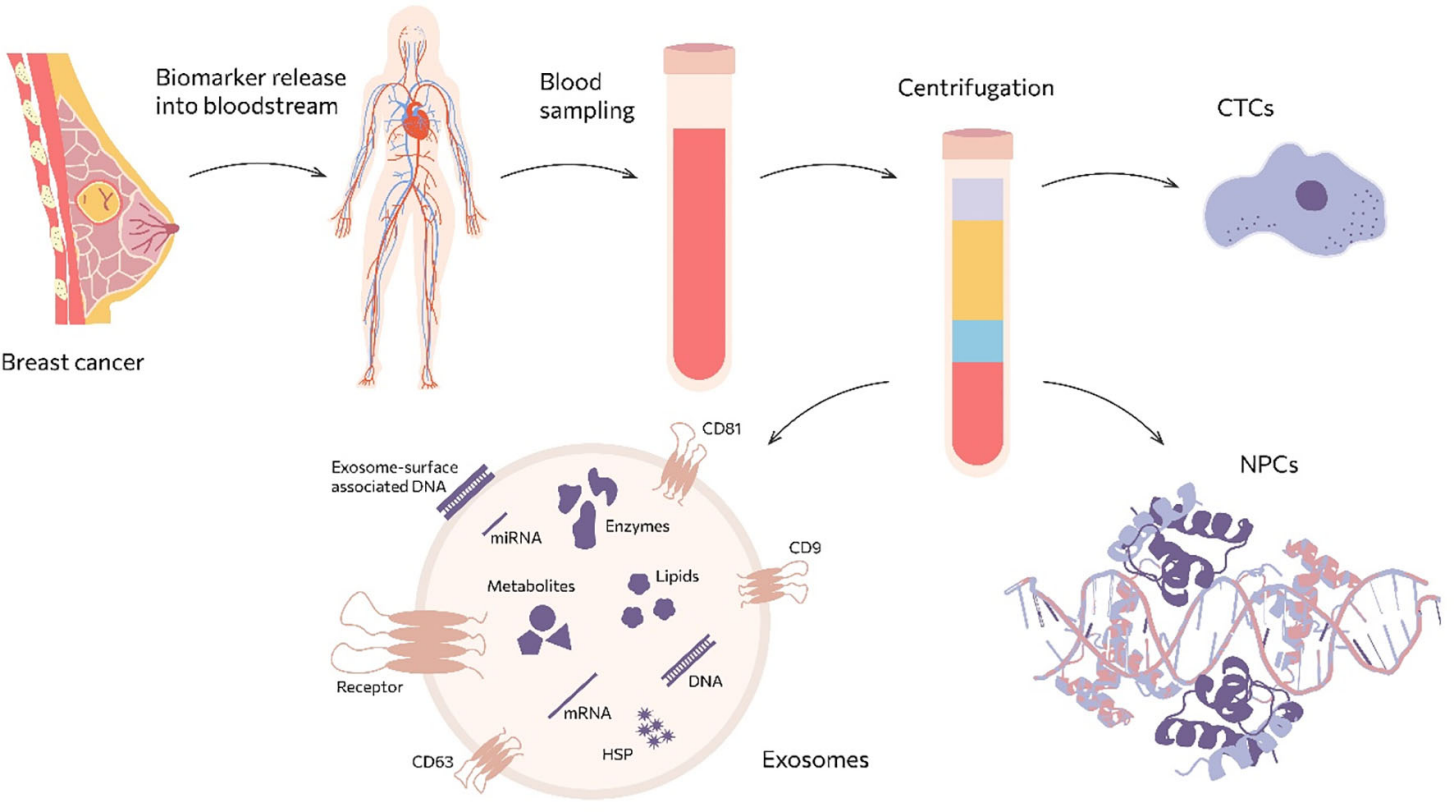
Extracellular vesicles (EVs) carry a complex and heterogeneous cargo comprising membrane-associated and luminal biomolecules, including proteins, nucleic acids, lipids, and metabolites. Surface-associated components, such as tetraspanins (CD9, CD63, CD81), receptors, immunoglobulins, and proteases (e.g., ADAMs and MMPs), reflect the cellular origin of EVs and are readily accessible for affinity-based isolation and detection strategies. In contrast, luminal cargoincluding mRNA, microRNA, DNA fragments, enzymes, heat shock proteins, lipids, and metabolitesencodes functional and metabolic information related to tumor biology and disease state. This schematic highlights the multimodal nature of EV-associated biomarkers and emphasizes why EVs represent a uniquely informative source for liquid biopsy, enabling simultaneous analysis of surface markers and internal molecular signatures.

According to current consensus definitions, extracellular vesicles are lipid−bilayer−enclosed particles of cellular origin that are unable to replicate independently (22). Exosomes represent a subpopulation of small EVs (~30–150 nm) formed as intraluminal vesicles within multivesicular bodies and released upon fusion with the plasma membrane (23). The biogenesis of exosomes, in contrast to other EVs, occurs through a multistep process. Initially, invagination of the plasma membrane leads to the formation of early endosomes. Subsequent inward budding of the endosomal membrane gives rise to intraluminal vesicles (ILVs) (24). The accumulation of ILVs within a single endosome result in the formation of multivesicular bodies (MVBs), which either fuse with lysosomes or autophagosomes for degradation or are transported to the plasma membrane, where fusion releases ILVs into the extracellular space as exosomes. The transformation of endosomes into multivesicular bodies (MVBs) can occur in both ESCRT-dependent and ESCRT-independent ways (23), but the fundamental principle remains the same: the proteins in the exosome “crown” reproduce the molecular profile of the parent cell (**Figure 2**). In general, the total exosomal cargo consists of various molecules, such as different classes of RNA, proteins, lipids, and metabolites (24). Regardless of their origin, all exosomes invariably contain molecules involved in their biogenesis, secretion, transport, and interaction with recipient cells.

The mechanisms that regulate exosome secretion are heterogeneous and depend on the type of parent cell and the specific cargo of the vesicle. Rab GTPases and SNAP/SNARE proteins are involved: Rab5 and Rab7 mediate cargo delivery to forming exosomes, while Rab27a and Rab27b promote MVB fusion with the plasma membrane. Ral-1, Rab11, and Rab35 also play a role in regulating exosome secretion (25).

There are several mechanisms by which exosomes interact with recipient cells (26):

- binding of exosome crown proteins to cell surface receptors without membrane fusion;
- direct fusion with the plasma membrane, resulting in the release of molecular cargo into the cell cytoplasm;
- internalization through endocytosis by the recipient cell.

It is known that universal exosome crown proteins include tetraspanins (CD9, CD63, and CD81), integrins, adhesion receptors, annexins, and flotillins. These proteins are all critical for recognizing and interacting with target cells (24, 27, 28), and are widely expressed across extracellular vesicle populations serving primarily as structural and normalization markers rather than tumor−specific indicators; their diagnostic utility lies in quantitative shifts or co−expression patterns rather than mere presence (29). Other common exosome crown proteins include matrix metalloproteinases (MMPs), whose levels increase significantly on the surface of tumor-derived exosomes (28). Beyond the interaction mechanisms listed above, exosomes employ diverse uptake pathways, including clathrin−dependent and clathrin−independent endocytosis, caveolin−mediated uptake, macropinocytosis, phagocytosis and direct membrane fusion. The choice of pathway depends on the repertoire of proteins and glycoproteins on both the exosome and the recipient cell (30).

## 3 The role of exosomes in breast cancer dissemination

Exosomes serve as active regulators of both physiological and pathological processes, including embryonic development, immune responses, tissue regeneration, blood coagulation, and angiogenesis (31, 32). Despite the rapid growth in the number of studies dedicated to EVs over the past decade, many aspects of intercellular communication via exosomes remain insufficiently understood. These include the dynamics and regulation of exosome secretion, mechanisms of intracellular molecular sorting and targeted delivery of their contents between cells.

### 3.1 The effects of tumour-derived exosomes on immune system functionality

Exosomes contribute to the formation of an immunoprivileged tumour microenvironment by transferring pro-apoptotic molecules, such as Fas ligand and TRAIL, to activated T lymphocytes (33–35). *In vitro* studies using cell lines have demonstrated that the molecular cargo of tumour-derived exosomes, including MICA/B, ULBP3, TGF-β, PI-9, and various microRNAs, modulates natural killer (NK) cell function by downregulating the surface receptor NKG2D. At the same time, the protease inhibitor PI-9 degrades granzyme B (36, 37). Furthermore, reduced levels of perforin in NK cell lytic granules have been linked to decreased LAMP-1 expression, which controls granule trafficking (38, 39).

Tumor-derived exosomes also affect the functional activity of antigen-presenting cells. In particular, these vesicles inhibit the differentiation of CD14^+^ monocytes into dendritic cells, thereby increasing the population of CD14^+^HLA-DRneg/low cells (40, 41).

Thus, by inducing an immunosuppressive state, tumor-derived exosomes facilitate immune evasion by malignant cells.

### 3.2 Stimulation of epithelial-mesenchymal transition, migration, and invasion by tumor exosomes

There is compelling evidence that the contents of tumour-derived exosomes, such as TGF-β, HIF-α, β-catenin, IL-6, caveolin-1, vimentin, and EMT-promoting microRNAs, participate in all stages of epithelial-mesenchymal transition (EMT), from acquisition of an invasive phenotype to establishment of distant metastases (42, 43). In particular, it was demonstrated in breast cancer (BC) that the transport of MMP-1 via exosomes promotes EMT and enhances metastatic dissemination (44). Comparative proteomic analyses of blood exosomes from healthy females and BC patients have revealed increased expression of EMT-stimulatory proteins in the latter group (45).

Exosomes are known to carry tetraspanins (CD9, CD81, CD82, CD151) (46, 47), as well as mature forms of ADAM-10 and ADAM-17 (48). CD151 regulates cell migration by modulating the activity of ADAM-10 and ADAM-17 (49), which hydrolyse the extracellular domains of transmembrane proteins, thereby modulating cell adhesion, migration, and intercellular interactions (50). In particular, ADAM-10 mediates the proteolysis of E-cadherin; the soluble form of E-cadherin disrupts cell-to-cell adhesion, promoting cell migration and increasing metastatic potential (46). In combination with TGF-β, CD151 enhances invasion and metastasis by activating signaling pathways, including Smad2/3, c-Akt, Erk1/2, JNK, Jun, and MMP-9 (51).

### 3.3 Stimulation of angiogenesis and metastasis by tumor-derived exosomes

Exosomes of tumor origin play an active role in the angiogenic processes that support neoplasia development. Specifically, MMPs at the exosomal crown are involved in degrading the basement membrane, facilitating capillary sprouting and the release of angiogenic factors (52). MMPs and tetraspanins have been identified on the surface of exosomes, promoting the expression of proangiogenic molecules, including VEGF, VEGFR1, VEGFR2, uPA, MMP-2, and MMP-9 (53).

Exosomes secreted by tumor cells play a pivotal role in metastasis by preparing premetastatic niches and attracting malignant cells to these sites, functioning as primary mediators in the formation of metastatic foci (54). These exosomes carry proteins such as ADAM-10, ADAM-17, Cadherin-11, Fibronectin 1, and Laminin gamma 1, all of which contribute to tumor growth and metastasis (55, 56).

Tumor cells typically exhibit organotropism, forming metastases in specific target organs. For example, triple-negative BC (TNBC) tends to metastasize to the lungs and brain, while other BC subtypes preferentially spread to bone and soft tissue. Tumor-derived exosomes carry organotropic proteins that direct metastasis: integrins (ITGα6β4 and ITGα6β1) are associated with lung metastasis, ITGαvβ5 is linked to liver tropism (57). Additionally, exosomal Annexin II promotes angiogenesis and metastatic progression in aggressive BC phenotypes (58), while MMP-1 transported in TNBC-derived exosomes enhances lung metastasis via PAR1 receptor activation (59). Moreover, CEMIP-enriched exosomes from brain metastatic cells induce inflammation and vascular remodeling in the brain microenvironment, establishing a permissive niche for tumor colonization (60).

Therefore, exosome crown proteins are crucial for intercellular communication in both the primary tumor microenvironment and distant metastatic foci.

### 3.4 Exosomes in context of liquid biopsy

Exosomes differ from other liquid−biopsy targets, such as circulating tumor cells and circulating tumor DNA, they are abundant, structurally stable and present in various body fluids (31). Unlike ctDNA, exosomes preserve intact proteins and nucleic acids that reflect the phenotype of the parental cell. Standardized kits allow rapid exosome isolation, and markers such as HSP70 and Alix enable quality control (61). However, high population heterogeneity and strong dependence on isolation methods complicate data interpretation. Consequently, exosomes permit multiparametric phenotyping and longitudinal monitoring but require methodological standardization and multiplexed panels to achieve clinical specificity (24, 25).

## 4 Exosome corona proteins as promising markers of BC

Because the exosome membrane is similar in composition to the parent cell membrane, a subset of surface and transmembrane proteins presents in tumor exosomes demonstrate high diagnostic and prognostic value, especially when evaluated by high-resolution flow cytometry (FCM). Some of these proteins have been directly identified by FCM in clinical or experimental settings, while others represent compelling targets for future FCM-based detection (62, 63).

According to Vesiclepedia (http://www.microvesicles.org), as of 20 February 2025, a total of 5,669,119 proteins had been catalogued in association with exosomes. The heterogeneity of the exosomal proteome is primarily attributed to the morphological features of the parent cells and the cellular conditions (e.g. heat shock or hypoxia) under which the vesicles are produced. Furthermore, the protein composition of the exosomal surface is influenced by the biological fluids in which the vesicles circulate, as well as by the techniques used for isolating and purifying them (24, 25).

**Figure 3:**
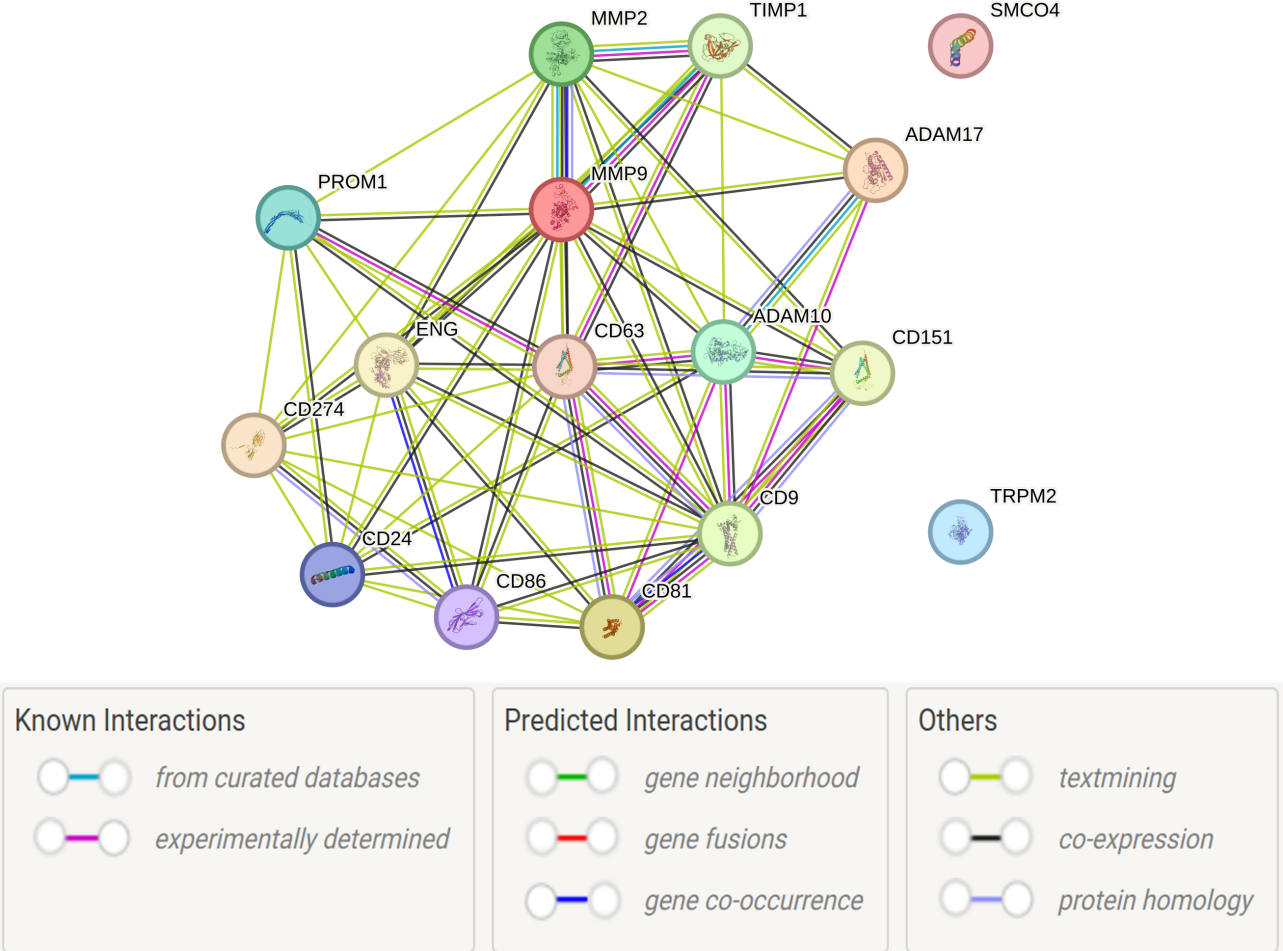
PPI of exosomal crown proteins identified in different cancer types.

Proteomic analysis of the most frequently identified exosomal crown proteins, conducted using the STRING v11 database platform (https://string-db.org/). STRING integrates physical and functional associations derived from curated databases, experiments, co−expression and text mining. A medium−confidence combined score (≥ 0.4) was selected to visualize functionally coherent modules among 14 of the 16 proteins. (**Figure 3**).

This high degree of interconnectivity suggests that most of these proteins are involved in shared molecular pathways that are implicated in tumor progression, immune modulation or cellular adhesion. This supports their biological relevance in oncogenic exosome biology. However, the STRING framework did not establish any direct protein–protein interaction (PPI) links for two transmembrane proteins: TRPM2 and SMCO4. The absence of network connectivity does not negate their biological significance, but rather suggests an under-characterized or independent functional axis.

TRPM2 (transient receptor potential melastatin 2) is a Ca²^+^-permeable, non-selective cation channel that is activated by oxidative stress and ADP-ribose. It has been implicated in redox homeostasis, inflammatory responses and tumor cell survival mechanisms, particularly in BC (64, 65).

SMCO4 (Single-Pass Membrane Protein with Coiled-Coil Domain 4) is a poorly characterized transmembrane protein. While its precise molecular function remains unclear, recent transcriptomic analyses have associated it with an unfavorable prognosis in certain BC subtypes, suggesting a potential role in oncogenic signaling or exosomal cargo regulation (59, 65).

STRING-based interactome mapping supports the notion that most exosomal surface proteins operate together within tumor-associated pathways. The apparent isolation of TRPM2 and SMCO4 within this network underscores the necessity of further functional annotation and experimental validation to clarify their roles in exosome biology and cancer progression.

In addition to the variable surface proteome influenced by external and intrinsic cellular factors, heat shock proteins (HSPs) are known to adsorb onto the surface of extracellular vesicles (EVs), where they serve a protective role for both the vesicles and their molecular cargo (58). These chaperone proteins help preserve the structural integrity of intracellular proteins and contribute to cellular protein homeostasis under both physiological and stress-induced conditions (58). In tumor vesicles, HSPs have been shown to suppress apoptotic signaling and reduce the efficacy of cytostatic and radiotherapeutic drugs.

### 4.1 Tetraspanins as critical components of exosomal membrane

Tetraspanins are a family of transmembrane proteins characterized by a conserved molecular structure consisting of four transmembrane domains, two extracellular loops (one small and one large), and short cytoplasmic tails. The large extracellular loop contains 4–8 conserved cysteine residues (55). These proteins typically undergo post-translational modifications, such as glycosylation of the extracellular loops and palmitoylation of the intracellular cysteines. They also contain sorting motifs that facilitate targeting to intracellular compartments and enable internalization via interactions with associated proteins (66).

Tetraspanins have a strong propensity to associate with one another, forming specialized membrane microdomains that interact with various transmembrane and cytosolic signalling proteins, including immunoglobulins, proteases, integrins (α2, α6, β1 and β4) and other proteins that are unique to exosomes, such as syntenin, TSG101 and ALIX (67, 68). Consequently, tetraspanins are involved in a variety of cellular processes, such as adhesion, the regulation of motility and morphology, membrane fusion and intracellular signaling.

Thus, changes in the expression of specific tetraspanins may affect the targeting and internalization of exosomes, ultimately influencing recipient cell behavior. *In vitro* and *in vivo* studies have demonstrated that exosomes circulate throughout the body and selectively bind to cells depending on the pairing of exosomal tetraspanins with cellular ligands (69). For instance, the combination of CD9 and CD81 tetraspanins with integrin αvβ3 directs exosomes to dendritic cells, whereas complexes of tetraspanin-8 and integrin CD49d (α4) facilitate the targeting of tumor-derived exosomes to endothelial cells (70). Another study found that antibody-mediated blockade of tetraspanins (CD9, CD81, CD151 and tetraspanin-8) reduced exosome uptake by recipient cells (71). Notably, some cellular responses can be triggered by exosomes even in the absence of internalization, as demonstrated in several studies (72, 73).

In human blood, the most commonly encountered exosomes contain CD9, CD63 and CD81. CD9 is a 15.91 kDa tetraspanin (74) that is expressed in a wide range of haematopoietic and non-haematopoietic cells, including epithelial, endothelial, stromal and various malignant cell types (75). As a typical member of the tetraspanin superfamily, CD9 participates in the formation of tetraspanin-enriched microdomains, thereby influencing key cellular processes such as intracellular signalling, proliferation, migration, invasion and adhesion (76).

The literature presents conflicting data regarding the functional role of CD9 in BC. Some studies suggest tumour-suppressive properties (77, 78), while others demonstrate a pro-metastatic role (79). CD9 expression has been shown to influence several stages of the metastatic cascade, including intravasation, colonization, and secondary tumour growth (80). Furthermore, CD9 may promote both tumour angiogenesis and lymphangiogenesis (74, 81). Through its association with E-cadherin and β-catenin, CD9 facilitates the export of β-catenin via exosomes, which can modulate Wnt signalling (79). Similarly, CD9 is involved in the incorporation of the metallopeptidase CD10 into exosomes (82).

CD63 is a membrane protein belonging to the tetraspanin superfamily with a molecular mass of 14.27 kDa (74). It is expressed on the surface of platelets and various immune cells, including activated monocytes, macrophages, granulocytes, lymphocytes (83), dendritic cells, and certain tumor cells. Since CD63 also localizes to endosomes, lysosomal membranes, and other intracellular compartments (84), it is considered a late endosomal, lysosomal, and exosomal marker protein. CD63 has been shown to interact directly or indirectly with a wide range of proteins, including integrins (LFA-1, α1-α6), cell surface receptors (FcRI, MHC-II, CD3), other tetraspanins (CD9, CD151, CD81, CD82), kinases (Lyn, Hck, phosphatidylinositol 4-kinase), adaptor proteins (AP-2, AP-3, AP-4), and exosome-specific proteins such as syntenin-1 and L6 antigen (85, 86).

These interactions enable CD63 to influence various cellular functions, including the remodelling of the actin cytoskeleton, platelet activation, cell adhesion and migration, and the intracellular trafficking and localisation of proteins (87–91). CD63 exhibits functionally ambivalent properties and may exhibit either tumour-suppressive or tumour-promoting activity, depending on the cancer type and microenvironment (92). For instance, the CD63-TIMP-1 axis has been demonstrated to encourage BC progression by inducing carbonic anhydrase IX expression, thereby acidifying the tumour microenvironment (93). Furthermore, CD63 expression positively correlates with HER2 status in BC tissues (94).

CD63 contributes to resistance against anticancer drugs. CD63^+^ cancer-associated fibroblasts (CAFs) mediate tamoxifen resistance in BC cells via the exosomal delivery of microRNA-22 (95). The drug resistance and invasive potential of BC cells are also influenced by CD63 glycosylation and co-regulation with MDR1 (96). In TNBC models, CD63-containing exosomes convert classical M1 macrophages into M2 macrophages, thereby creating a favorable niche for lymph node metastasis (97). Notably, treatment with anti-CD63 antibodies has been shown to significantly reduce metastases to the chest cavity, lymph nodes and lungs (98).

CD81 is a 17.96 kDa (68) tetraspanin widely distributed on cellular membranes. It plays an important role in membrane organisation, protein trafficking, membrane fusion and cell-to-cell interactions (99, 100). CD81 also regulates cellular migration and invasion, thereby contributing to cancer progression. This molecule is overexpressed in many types of cancer, including breast, lung, prostate, melanoma, brain tumours and lymphomas, and its expression correlates with disease prognosis (99). In BC mouse models, tumour-associated fibroblasts (TAFs) secrete exosomes enriched in the tetraspanins CD63, CD81 and CD82. Of these, only CD81 is responsible for the exosomal incorporation of Wnt11. These vesicles are internalised by BC cells, where Wnt11 enhances migratory capacity and promotes metastasis (100).

Thus, CD9, CD63, and CD81 are not only critical for exosome formation and cargo selection but also for targeting recipient cells and activating downstream signaling pathways that drive tumor growth and metastasis.

In addition to canonical tetraspanins, emerging evidence suggests that several transmembrane proteins may serve as exosomal biomarkers in oncological settings. Notably, TRPM2 (transient receptor potential melastatin 2), SMCO4 and CD86 have been associated with BC via transcriptomic and proteomic profiling and may indicate PANoptosis-related immune modulation and patient survival (58). While these proteins have not yet been extensively validated via flow cytometry in exosomal contexts, their membrane localisation makes them plausible candidates for such detection methodologies. TRPM2, which is a Ca²^+^-permeable channel involved in redox signalling, and CD86, which is a co-stimulatory molecule found on antigen-presenting cells, could feasibly be detected using fluorophore-conjugated monoclonal antibodies in multiparameter FCM assays targeting exosomal subpopulations (58).

Additional studies have documented PD-L1 (Programmed Death-Ligand 1) as an exosomal transmembrane protein with key immunosuppressive functions. With a molecular weight of 21–33 kDa, its presence on circulating exosomes from tumor patients correlates with disease burden and immune evasion. Quantification via FCM has proven feasible in gastric cancer and BC, suggesting strong translational potential (101).

Another transmembrane tetraspanin, CD151, has gained attention for its role in cancer progression, including cell adhesion, migration, and metastasis. With a molecular weight of 28 kDa, CD151 has been identified on the surface of exosomes derived from BC cells, and its expression has been associated with unfavorable clinical outcomes (99). Its involvement in integrin-mediated signaling and interaction with other tetraspanins (CD9, CD81) underscores its diagnostic relevance and technical feasibility for FCM-based detection in exosomal preparations (8, 86).

Furthermore, FCM was employed to detect and quantify CD105 and CD133 - markers of angiogenesis and cancer stemness, respectively (101). These markers were co-expressed with CD81 on exosomes isolated from MDA-MB-231 cells, providing a multi-dimensional phenotypic profile that could predict aggressive disease and resistance to therapy (94). Taken together, these findings reveal a two-tier classification of exosomal proteins: those already detectable by FCM (CD63, CD81, PD-L1, CD151, CD105, CD133) and promising candidates for future implementation in FCM protocols [TRPM2, SMCO4, CD86) (8, 96–101)]. Integrating these protein targets into clinical FCM workflows will depend on ongoing efforts to standardize exosome isolation, antibody validation and signal amplification strategies.

### 4.2 Proteases in the crown of exosomes

Compared to other types of EVs, a distinctive feature of exosomal molecular cargo is the high proportion of enzymatic proteins, which can comprise up to 32% of total exosomal proteins (102, 103). Exosomal proteases can be classified into three categories:

- tetraspanin-associated proteases, which are structurally and functionally linked to tetraspanins;
- tetraspanin-independent proteases;
- proteases of undefined localization within EVs (104).

The best-characterized tetraspanin-associated proteases at exosomal crown are members of the ADAM (a disintegrin and metalloproteinase) and MMP (matrix metalloproteinase) families. It is important to note that exosomes do not autonomously secrete proteases. Instead, they may harbour active or pro−enzyme forms of proteases and can stimulate protease secretion in recipient cells; purified EVs themselves contain minimal MMP−9 but act as potent stimulators of MMP−9 secretion (44).

ADAMs are a family of transmembrane and secreted proteins consisting of approximately 750 amino acids. Only 12 of the known ADAMs exhibit proteolytic activity: ADAM-8, ADAM-9, ADAM-10, ADAM-12, ADAM-15, ADAM-17, ADAM-19, ADAM-20, ADAM-21, ADAM-28, ADAM-30 and ADAM-33. These proteases function as sheddases, cleaving the ectodomains of surface receptors and signaling molecules. These proteases are involved in numerous physiological processes; however, in cancer, specific ADAMs enhance tumour aggressiveness by stimulating proliferation through EGFR activation and promoting EMT by cleaving E-cadherin (105).

ADAM-10 and ADAM-17, the most extensively studied sheddases, have both been identified in exosomes (104). Notably, ADAM-10 has been shown to associate with CD9 and CD81 in exosomes (44, 105). Its substrates include membrane-bound cytokine, chemokine and growth factor precursors (e.g. CXCL16, EGF), adhesion molecules (e.g. N-cadherin, E-cadherin, VE-cadherin and CD44) and Notch receptors (106). It was demonstrated that the level of ADAM-10 increased in the plasma and total blood exosomes of BC patients compared to healthy females (107). In HER2-positive BC, targeted therapy with trastuzumab (a monoclonal anti-HER2 antibody) is standard, but disease relapse with increased resistance frequently occurs (108). Interestingly, trastuzumab therapy has been found to be associated with elevated ADAM-10 levels, and HER2 itself serves as a substrate for ADAM-10. The proteolysis of HER2’s extracellular domain by ADAM-10 yields a soluble form of the receptor, the level of which correlates with decreased progression-free survival (109). Furthermore, ADAM-10 and ADAM-17 overexpression has been observed in the majority of TNBC cases (110). *In vitro*, RNA interference (RNAi) knockdown or chemical inhibition of ADAM-10 (e.g. with GI254023X) reduces the migratory capacity of TNBC-like cells, while ADAM-10 re-expression restores their proliferation, migration, and invasion potential (109).

ADAM-17 and ADAM-10 have many shared substrates due to their similar specificity (111). Numerous studies indicate that elevated ADAM-17 expression in BC is associated with increased invasiveness and resistance to therapy. This protease promotes tumour progression by stimulating cell proliferation, invasion, angiogenesis and trastuzumab resistance (112).

MMPs are a family of zinc-dependent endopeptidases that play a crucial role in extracellular matrix (ECM) degradation (113). Like ADAMs, MMPs are involved in physiological processes such as tissue remodelling, inflammation, and wound healing. In malignancy, they facilitate tumor growth, invasion and metastasis by degrading basement membranes and ECM components (114), while also releasing proangiogenic factors (e.g. VEGF) that enhance tumor vascularization (115).

In the blood-derived exosomes of cancer patients, MMP-2 and MMP-9 are the most well-studied (116). Elevated levels of exosomal MMP-9 have been reported in osteosarcoma patients (117) and in exosomes from pancreatic cancer cell lines (116). Moreover, EVs from the blood of TNBC patients increase migration and invasion, as well as secretion of MMP-2 and MMP-9 in MDA-MB-231 cells (a TNBC model) (118). Similarly, lung cancer patient-derived exosomes enhance MMP-9 secretion in A549 adenocarcinoma cell (119). Moreover, several recent studies have reinforced the significance of exosomal proteases in tumor progression and their utility as biomarkers in BC. Notably, exosomes derived from the plasma of women with metabolically unhealthy phenotypes regardless of BMI exerted pronounced pro-tumorigenic effects on MDA-MB-231 TNBC cells. These effects included enhanced cell migration, invasion, and elevated MMP-2 activity (120). In contrast, exosomes from metabolically healthy normal-weight individuals exhibited reduced invasiveness. Interestingly, MMP-9 activity was not significantly altered among the groups, suggesting a differential regulation of gelatinases by obesity-related EVs (120). The functional role of MMPs in EMT and tumor aggressiveness is further supported by studies showing that inhibition of the MAPK/ERK signaling cascade in TNBC cells reduces MMP-2 and MMP-9 expression and activity. In one study, exosomes were used as delivery vehicles for anti-MEK1 siRNA (iExoMEK1), leading to effective downregulation of the MAPK/ERK pathway and associated MMP expression. This resulted in the reversion of mesenchymal features, suppression of cell migration and invasion, and decreased angiogenesis in TNBC models (121). In addition to MMP-2 and MMP-9, emerging data suggests that exosomal MMP-1 may also play a relevant role in early detection of BC. A study evaluating urinary exosomes reported that combined detection of miR-21 and MMP-1/CD63 could identify 95% of early-stage, non-metastatic BC cases. Specifically, MMP-1/CD63 levels were significantly higher in BC patients compared to healthy controls, highlighting the utility of urinary exosomal protease profiling as a non-invasive screening method (122). Additionally, recent studies have further elucidated the specific contributions of exosomal MMP-1 to TNBC metastasis. Exosomes isolated from a highly pulmonary metastatic variant of MDA-MB-231 cells (MDA-MB-231-HM) were shown to be enriched in MMP-1 and capable of transferring this aggressive phenotype to less metastatic TNBC cells, such as parental MDA-MB-231, MDA-MB-468 and BT-549 (41). Following uptake, recipient cells displayed enhanced migratory and invasive behavior alongside increased endogenous MMP-1 secretion. Mechanistically, exosomal MMP-1 was found to engage protease-activated receptor 1 (PAR1), a G-protein-coupled receptor, triggering EMT and thereby amplifying metastatic potential. Inhibition of PAR1 with the antagonist vorapaxar significantly reversed these pro-metastatic effects, confirming the functional relevance of the MMP-1/PAR1 axis in exosome-mediated TNBC progression (41). These findings are supported by complementary evidence from patient-derived exosomes. Plasma-derived EVs from women with BC, especially those with TNBC, significantly increased the migratory and invasive capacity of MDA-MB-231 cells, accompanied by elevated secretion of MMP-2 and MMP-9. This effect was shown to depend on Src-kinase activation and downstream focal adhesion assembly, as inhibition of Src with PP2 reduced EV-induced invasion. Importantly, these effects were independent of hormone receptor and HER2 status, suggesting a generalized mechanism of EV-driven ECM remodeling and invasion in TNBC (119).

Together, these findings underscore the multifaceted role of exosomal proteases, particularly MMP-1, MMP-2 and MMP-9, in tumor progression, invasion, and diagnostics. Their activity is tightly linked to upstream signaling pathways such as MAPK/ERK and is modulated by host metabolic status, suggesting that EV-associated proteases represent both functional effectors of tumor aggressiveness and promising targets for therapeutic or diagnostic intervention in BC.

### 4.3 Catalytic antibodies on exosomal surface

Although the association between immunoglobulins, particularly immunoglobulin G (IgG), and EVs remains incompletely understood -partly due to the possibility of methodological artifacts and the complexity of post-secretory molecular interactions, emerging evidence necessitates consideration of these interactions to fully elucidate the role of EVs in tumor biology and therapy response.

Multiple studies have documented that IgG isolated from the serum of patients with gastric and thyroid carcinomas exhibit DNA-hydrolyzing activity (123). Similar catalytic properties have been reported in IgG derived from individuals with lymphoproliferative disorders, including lymphomas and chronic lymphocytic leukemia (124). More recently, mass spectrometric analysis has identified IgG as a constituent of the protein corona of EVs, suggesting a potentially active functional role for these immunoglobulins within the vesicular context (125). In addition to nuclease-like activity, peroxidase and catalase functions have been attributed to serum IgG and IgA in subsets of BC patients (126) and to IgG in gastric cancer patients (127), indicating that vesicle-associated IgG may similarly retain enzymatic activities under oncological conditions. These findings support the hypothesis that vesicle-bound immunoglobulins could contribute to tumor-associated oxidative stress regulation or act as unconventional enzymatic effectors.

Notably, elevated levels of IgG-positive EVs (IgG+EVs) have been reported in patients with pancreatic ductal adenocarcinoma, reinforcing earlier suggestions of their potential utility as diagnostic markers for this malignancy (128). Moreover, quantitative analysis has demonstrated a correlation between increased IgG+EV abundance and lack of response to chemotherapy, whereas a marked reduction in these EVs has been observed in chemotherapy-responsive pancreatic ductal adenocarcinoma. Importantly, the concentration of IgG+EVs did not correlate with total serum IgG levels or classical systemic inflammation markers, such as the neutrophil-to-lymphocyte ratio and C-reactive protein, indicating that IgG+EV abundance is not substantially influenced by the inflammatory status of patients (128).

It is proposed that a subset of IgG molecules may bind to EVs through post-secretory protein–protein interactions, possibly mediated by surface-exposed vesicular proteins such as melanoma-associated antigen B1 (MAGE B1) (129). This interaction appears to occur in both healthy individuals and cancer patients. Moreover, recent studies have challenged the long-held view that immunoglobulin production is restricted to plasma cells by demonstrating *de novo* synthesis of IgG in various tumor types (130, 131). These findings suggest that a portion of circulating IgG+EVs may originate from direct vesicular secretion by tumor cells, rather than through peripheral IgG adsorption alone.

Thus, despite the fact that association between IgGs and EVs remains vague, the available data underscore the potential multifaceted role of IgG in cancer pathophysiology and therapy monitoring. While mechanistic ambiguities persist regarding the origin and functional integration of IgG within EV populations, their consistent association with enzymatic activity, tumor progression, and treatment response highlights their diagnostic and prognostic potential. Further elucidation of the mechanisms governing IgG-EV interactions may provide novel insights into tumor biology and support the development of IgG+EVs as clinically relevant biomarkers in oncology.

## 5 Conclusion

The results presented here highlight the high potential of exosomal coronary proteins as molecular markers for diagnosis, prognosis and therapeutic monitoring of BC. Surface-associated proteins, including tetraspanins (CD9, CD63, CD81, CD151), proteases (ADAM-10, ADAM-17, MMP-1, MMP-2, MMP-9), novel transmembrane proteins (TRPM2, SMCO4, CD86) and antibodies, demonstrate significant diagnostic specificity and functional relevance in key oncogenic processes such as angiogenesis and immune surveillance. Multiparameter flow cytometry combined with high-affinity fluorophore-conjugated antibodies represents a robust and clinically applicable platform for the detection of these exosomal proteins. Standardization of protocols for exosome isolation, antibody validation, and signal amplification will be critical for the successful implementation of this approach into routine clinical practice. Future studies should prioritize deep functional annotation of understudied exosomal proteins such as TRPM2 and SMCO4 and the creation of combinatorial marker panels with integrated diagnostic, prognostic, and therapeutic value. The integration of exosomal coronary protein profiling into modern oncology workflows may open new opportunities for early detection, long-term surveillance, and personalized treatment of BC.
